# The Impact of Early Pregnancy and Exposure to Tobacco Smoke on Blood Antioxidant Status and Copper, Zinc, Cadmium Concentration—A Pilot Study

**DOI:** 10.3390/antiox10030493

**Published:** 2021-03-22

**Authors:** Anna Bizoń, Halina Milnerowicz, Katarzyna Kowalska-Piastun, Ewa Milnerowicz-Nabzdyk

**Affiliations:** 1 Department of Biomedical and Environmental Analysis, Wroclaw Medical University, 50-556 Wroclaw, Poland; halina.milnerowicz@umed.wroc.pl (H.M.); katarzyna.ew.kowalska@gmail.com (K.K.-P.); 2 2nd Department and Clinic of Obstetrics and Gynecology, Wroclaw Medical University, 50-556 Wroclaw, Poland; ewa.milnerwoicz@wp.pl

**Keywords:** antioxidants status, early pregnancy, exposure to tobacco smoke

## Abstract

The aim of the study was to evaluate the impact of early pregnancy and exposure to tobacco smoke on antioxidant status and copper, zinc, and cadmium concentrations in the blood of non-smoking and smoking, as well as non-pregnant or pregnant women. The study included 213 women. More specifically, 150 women in first trimester of pregnancy and 63 non-pregnant women. Women were divided into subgroups according to exposure to tobacco smoke. Pregnancy significant influences higher copper and lower zinc concentration in the serum, whereas exposure to tobacco smoke during pregnancy is mainly associated with an elevation in cadmium and zinc concentration. It seems that metallothionein, superoxide dismutase, and glutathione peroxidase are the important antioxidants during early pregnancy, when exposure to tobacco smoke occurs, whereas the pregnancy itself is associated with a higher concentration of metallothionein and activity of catalase. Both pregnancy in the first trimester and exposure to tobacco smoke decrease glutathione concentration. In addition, active and passive maternal smoking have a similarly negative effect on antioxidant status in the first trimester. Early pregnancy as well as exposure to tobacco smoke is associated with significant alteration in antioxidant status and copper, zinc, and cadmium concentration. Due to a small number of smoking subjects (11 cases of non-pregnant, active smokers and 14 pregnant active smokers), the obtained results should be treated as a pilot, and this should be considered for future studies.

## 1. Introduction

Pregnancy is a physiological state associated with increased metabolism and demand for oxygen, which can cause excessive production of reactivity oxygen species (ROS) and deficiency of antioxidants [1]. Oxidative stress has been associated with many reproductive and pregnancy disorders, including pregnancy loss [2,3]. Between 10 and 12 weeks of gestation, the trophoblast plugs are dislodged from maternal spiral arteries, filling the intervillous space with maternal blood [4]. This is accompanied by a sharp rise in oxygen tension, marking the establishment of full maternal arterial circulation to the placenta associated with an increase in ROS and oxidative stress [5]. Exposure to tobacco smoke during pregnancy intensifies metabolic and biochemical disorders and adaptive responses in both the fetus and the mother [6]. The impact of tobacco smoke on fetal growth may be observed from the beginning of pregnancy [7]. In our earlier study, we revealed that the trophoblast was significantly smallest in smoking pregnant women and its lower volume was correlated with higher cotinine and cadmium (Cd) concentration [7]. In addition, chronic exposure to tobacco smoke during pregnancies complicated by intrauterine growth restriction (IUGR) impaired flows in fetal arteries and veins and increased the risk of hypoxia in decompensation phase [8]. Furthermore, we observed in the group of women with diagnosed oligohydramnios or premature rupture of membranes that exposure to tobacco smoke altered the distribution of Cd, lead (Pb), and zinc (Zn), which probably affected placental tissue and fetal membranes [9].

The smoking pregnant woman exposes her fetus to a variety of harmful chemicals [10] which disturb, inter alia, the homeostasis of trace elements, such as Zn and Cu [11]. Previous studies showed that the optimum concentration of Zn and Cu, as well as the correct value of the Cu/Zn ratio, plays a crucial role in fertility and reproductive outcomes [12]. Physiological and metabolic changes of pregnant women as well as increased demands for the important elements such as Zn and Cu by the developing fetus, decrease their concentrations in maternal blood, and lead to pregnancy complications [13,14]. Zn is an essential trace element required by the human body for a variety of biological functions and is required for reproduction and promotes healthy growth [13]. While Zn deficiency was associated with a lower implantation rate, abnormal ovarian development, ovarian follicular growth, and oocyte maturation [15], too high Cu concentrations are associated with the promotion of oxidative stress [16]. Contemporary medicine indicates that lower maternal plasma Cu concentrations in the first trimester of pregnancy is a better protection against risk for any pregnancy complication when compared with women with high plasma Cu [17,18]. It was also claimed that Cu may be more important for placentation rather than conception [17]. In the first trimester of pregnancy, antioxidants protect trophoblast cells against oxidative stress [16,19]. One of the important non-enzymatic antioxidants is metallothionein (MT), a low molecular weight protein which mainly scavenges O_2_^•−^ or ^•^OH [20]. MT binds, but also is induced by, both essential (for example Zn and Cu) and toxic metals (for example Cd) [21]. The presence of MT was demonstrated in the human placenta and fetal membranes [22,23,24]. Furthermore, glutathione (GSH) is also one of the first-line non-enzymatic antioxidants which detoxify O_2_^•−^ or ^•^OH [25]. It was shown that Zn deficiency is accompanied by an increase in oxidants which decreases GSH concentration [20]. Cu and Zn are also required for superoxide dismutase (SOD) activity, a universal antioxidant enzyme, which catalyzes O_2_^•−^ to hydrogen peroxide (H_2_O_2_) and molecular oxygen (O_2_) [26]. Hydrogen peroxide is still highly toxic, but less reactive than O_2_^•−^ [27] and is further converted by catalase (CAT) or selenium-dependent glutathione peroxidase (GPx) to water (H_2_O) [28].

Alterations in nonenzymatic and enzymatic antioxidants during pregnancy was demonstrated in many investigations. Changes in MT concentration could be associates with disorders in homeostasis of Zn and Cu as well as detoxification of Cd both in maternal and umbilical cord blood, which could negatively affect the fetus development [1,29,30]. The drop in SOD activity was found during pregnancy complicated by preeclampsia [31], IUGR (1), in the blood of women with recurrent miscarriage [14] or missed abortion [32]. Moreover, reduced activity of GPx during pregnancy could be associated with hypertension of preeclampsia [33] and spontaneous abortion [34], while decreased activity of CAT was proposed as a promising predictor for preeclampsia [35].

To sum up, our study aimed to assess the effect of pregnancy and exposure to tobacco smoke during the first trimester of pregnancy on the status of non-enzymatic- or enzymatic antioxidants and Zn, Cu, and Cd concentrations in the blood of women. Our investigations used blood samples from non-pregnant women and women in the first trimester of pregnancy, exposed and not exposed to tobacco smoke.

## 2. Materials and Methods

The study included 150 women in weeks 11 to 14 of pregnancy: 52 of them were exposed to tobacco smoke (38 passive tobacco smokers and 14 active tobacco smokers) and 98 were non-smoking healthy pregnant women without any complications during pregnancy. The main inclusion criterion was singleton pregnancy. Women with any complications during their current or previous pregnancy (for example, diabetes, pregnancy-induced hypertension, preeclampsia, fetal or uterine malformations; pregnancies resulting from infertility treatment, vaginal bleeding) were excluded from the study. Control subjects were 63 young non-pregnant women, not exposed to tobacco smoke (*n* = 52) or actively smoking (*n* = 11). Pregnant women were of similar age and had similar BMI values. Women from the control group also were of similar age and had similar BMI values. The characteristics of pregnant women and the control group were summarized in Table 1. Pregnant and non-pregnant women were divided into subgroups according to cotinine (metabolite of nicotine) concentration and information was obtained in a personal interview. Biochemical parameters of both the study group and the control group were assayed in the Department of Biomedical and Environmental Analysis, Wroclaw Medical University. Blood collection, as well as clinical investigations of pregnant women, were performed in the 2nd Department and Clinic of Obstetrics and Gynaecology, Wroclaw Medical University. The study was approved by the Local Bioethics Committee of Wroclaw Medical University (KBN-152/2015). All pregnant women had undergone the prenatal examination in weeks 11–14 according to Fetal Medicine Foundation guidelines and were qualified for prenatal examination due to the mother’s age. The inclusion criteria, detailed characteristics, and conducted procedures including 3D ultrasound examinations and Doppler flow imaging were published earlier [7]. Also, in the control group, women taking any medicines including oral contraception/supplements were excluded from the study.

Cotinine concentration was assayed using a commercially available test (ref. No. 40-101-325056; GenWay Biotech Inc, San Diego, CA, USA). Serum Zn and Cu concentrations were determined with the use of flame atomic absorption spectrometry using SOLAAR M6, Thermo Elemental Co. Cd in whole blood was assayed using graphite furnace atomic absorption spectroscopy (GFAAS), with an absorbency measured at λ = 228.8 nm, using Zeeman background correction. Elements were assayed in a certified Atomic Absorption Spectroscopy Laboratory of the Department and Clinic of Internal and Occupational Diseases, Wroclaw Medical University.

The concentration of MT in plasma and erythrocyte lysate was measured by the two-step direct enzyme-linked immunosorbent assay (ELISA) using the method developed in our laboratory [36]. The concentration of GSH in whole blood was determined using a spectrophotometric method based on the reaction of GSH with alloxan and the formation of a complex with an absorbance maximum at 305 nm [37]. Superoxide dismutase activity in plasma was determined with Superoxide Dismutase Assay Kit (ref. No. 706002, Cayman Chemicals, USA). Plasma catalase activity was measured using the Catalase Assay Kit (ref. No. 707002, Cayman Chemicals, Ann Arbor, MI, USA). The assay of GPx activity in plasma was performed with the Glutathione Peroxide Assay Kit (ref. No. 703102, Cayman Chemicals, Ann Arbor, MI, USA).

Statistical analysis was performed using the Statistica software package, version 13.3 (Polish version; StatSoft, Kraków, Poland). Values were shown as mean ± SD and 1st quartile, median, 3rd quartile. The normality of the variables was tested with the use of the Shapiro-Wilk test. The homogeneity of variance was assessed using Levene’s test. The Student’s t-test was used when the normality of distribution and equality of variance were satisfied. The non-parametric Mann–Whitney U test was used when a lack of normal distribution and variance uniformity were revealed. Correlation was checked using the Pearson product-moment correlation coefficient or Spearman’s rank-order correlation coefficient. The value of *p* lower than 0.05 was considered significant.

## 3. Results

### 3.1. The Impact of Early Pregnancy on Metal Concentration and Antioxidant Status

#### 3.1.1. Metal Concentrations

A significantly lower Zn concentration was found in the group of non-smoking pregnant women when compared to the non-smoking control group. Cu concentration, as well as the value of Cu/Zn ratio, in both non-smoking and actively smoking pregnant women was significantly higher than in both groups of non-pregnant women. A higher Cd concentration was observed in the group of non-smoking pregnant women when compared to non-smoking, non-pregnant women. Zn and Cd concentrations were similar in both groups of actively smoking non-pregnant and pregnant women (Table 2 and Table 3).

#### 3.1.2. Non-Enzymatic Antioxidant Status

In the group of pregnant smoking women, GSH concentration was the lowest and almost 5-fold lower when compared to smoking non-pregnant women. Also, GSH concentration in the group of non-smoking pregnant women was lower when compared to non-smoking non-pregnant women. Plasma MT concentration in both groups of pregnant women was higher than in both groups of non-pregnant women, while MT concentration in erythrocyte lysate was similar in non-smoking pregnant women and non-smoking non-pregnant women (Table 2 and Table 3).

#### 3.1.3. Enzymatic Antioxidant Status

Significantly lower plasma SOD activity was revealed in both groups of pregnant women when compared to non-pregnant women (Table 2 and Table 3). Higher plasma CAT activity and lower GPx activity were found in the group of non-smoking pregnant women when compared to non-smoking non-pregnant women, whereas lower plasma CAT activity was observed in the group of actively smoking pregnant women when compared to actively smoking non-pregnant women (Table 2 and Table 3).

### 3.2. The Impact of Exposure to Tobacco Smoke during the First Trimester of Pregnancy on Copper, Zinc, and Cadmium Concentration and Blood Antioxidant Status

#### 3.2.1. Metal Concentrations

We revealed a statistically significantly higher serum Zn and whole blood Cd concentrations in the group of pregnant women exposed to tobacco smoke when compared to non-exposed pregnant women. Active exposure to tobacco smoke further increased Zn and Cd concentrations. Cu concentration was similar in all pregnant women. A lower value of Cu/Zn ratio was found in the group of pregnant women exposed to tobacco smoke when compared to non-exposed pregnant women (Table 4 and Table 5).

#### 3.2.2. Non-Enzymatic Antioxidant Status

Significantly higher plasma and erythrocyte lysate MT concentrations and lower GSH concentrations were found only in the group of pregnant women exposed to tobacco smoke when compared to the non-exposed group. The type of exposure to tobacco smoke did not influence MT and GSH concentrations (Table 4 and Table 5).

#### 3.2.3. Enzymatic Antioxidant Status

In the group of exposed pregnant women, significantly higher plasma SOD and GPx activity, and lower CAT activity was observed when compared to non-exposed pregnant women. The activity of SOD, GPx, and CAT was similar in both groups of passively and actively smoking pregnant women (Table 4 and Table 5).

### 3.3. Correlation Coefficient

Correlation coefficients in the group of non-smoking, passively, and actively smoking pregnant women are summarized in Table 6.

#### 3.3.1. In Non-Smoking Pregnant Women

Increased lysate MT concentration was associated with higher concentrations of Cu and higher values of the Cu/Zn ratio. GSH concentration was positively correlated with GPx activity as well as negatively with CAT activity.

#### 3.3.2. In Passively Smoking Pregnant Women

A negative correlation between GSH concentration and activity of CAT was observed. In addition, GPx activity was negatively correlated with Cu concentration, whereas higher CAT activity was associated with lower Zn concentration.

#### 3.3.3. In Actively Smoking Pregnant Women

GSH concentration was positively correlated with the activity of SOD or GPx. In addition, increased SOD activity was associated with higher Zn concentration, whereas a lower GSH concentration was associated with higher values of the Cu/Zn ratio.

## 4. Discussion

Little is known about the impact of pregnancy, especially in the first trimester, and exposure to tobacco smoke on antioxidant status in pregnant women, therefore in our study we assessed the levels of non-enzymatic (MT, GSH) and enzymatic antioxidants (SOD, CAT, GPx) in the blood of non-smoking and smoking women who were not pregnant or in the first trimester of pregnancy. In addition, we evaluated the influence of those factors on Zn, Cu, and Cd concentrations in the blood of the investigated women.

Regardless of exposure to tobacco smoke, higher concentrations of Cu and values of Cu/Zn were found in the serum of non-smoking and smoking pregnant women when compared to non-smoking and smoking non-pregnant women, respectively. Our results clearly show that pregnancy itself is associated with higher Cu concentration, whereas exposure to tobacco smoke, independently of type (passive or active smoking), did not influence Cu concentrations in the pregnant group, which coincides with our earlier study conducted in the group of pregnant women with IUGR [1] and with results obtained by other authors [10]. In addition, similar to our previous study, the average Cu concentration in the serum of pregnant women was higher than the reference range of the used method [1].

Increased Cu concentrations in pregnancy was also demonstrated by other authors [16], which was explained by an increase in its carrier proteins, ceruloplasmin, and elevated levels of maternal estrogens. It was also shown that, in serum of women with normal fetal development, Cu concentration was increased when compared to women with fetal developmental disorders [38]. On the other hand, it was demonstrated, that women at early stage of pregnancy with lower plasma Cu concentrations were better protected against risk for any pregnancy complication when compared with women with high plasma Cu [17], because higher concentration of Cu is associated not only with oxidative stress, but also with inflammation.

It was well documented that higher concentration of Cu could have teratogenic consequences [39] due to excessive cellular oxidative damage [40]. Oxidized reactions are greatly accelerated by the presence of Cu in the Fenton reaction [38]. Copper is one of the most important transition metals involved in the production of ^•^OH [16,38]. In the presence of a higher H_2_O_2_ concentration (especially when a lower activity of CAT or GPx occurs), Cu can generate ^•^OH, which is the most harmful free radical in the human body. In addition, a higher concentration of H_2_O_2_ can release iron ions from a range of haem proteins and further increase the production of ^•^OH and enhance oxidative stress.

In the case of Zn concentration, we revealed a significant drop in the serum of pregnant women when compared to that of non-pregnant women, but this finding was only observed in the group of non-exposed women. The literature has also shown lower Zn concentrations in the serum of pregnant women when compared to non-pregnant women [14,41]. During pregnancy, there is a drop in circulating Zn and a decrease also occurs as the pregnancy progresses, possibly due to a decrease in Zn binding and increased transfer of Zn from the mother to the fetus [41]. In addition, Zn does not undergo redox changes and as a consequence, it cannot be involved in electron transfer reactions, which is why the cellular toxicity of Zn is lower than that of Cu [42].

When we take into consideration the concentration of Zn only in the group of pregnant women, we found concentration in the group of smoking women to be significantly higher (with the highest value in the group of active smokers) than in the non-smoking women, which presumably could be associated with the presence of Zn in cigarettes [43]. The differences in Zn and Cu concentrations could be also associated with the age of pregnant women. The main features of pregnant women involved in our study were physiological single pregnancy and maternal age around 35 years old. The literature also indicates that aging is associated with decreased Zn status and an increased value of the Cu/Zn ratio [44], which can also explain the lower Zn concentration and higher value of the Cu/Zn ratio in our group of pregnant women.

In our study higher concentration of Cd was associated not only with exposure to tobacco smoke but also with pregnancy. In addition, active exposure to tobacco smoke in the first trimester increased Cd concentration more than passive exposure. Previous studies showed that higher Cd concentration in maternal blood was associated with decreased birth weight [45,46] and this effect was independent of cotinine-defined smoking status [47]. Furthermore, in the group of smokers, higher Zn concentration can protect against Cd toxicity [48] and those metals compete for the same binding targets, and Zn ions can reduce the adverse effect of Cd due to Zn induction of MT synthesis [49]. Increased placenta concentrations of Cd, Zn [23,50], and MT was earlier reported in smoking pregnant women [50]. In addition, MT is involved in the cellular storage and/or delivery of Cu ions to cuproenzymes [51], which potentially explains a positive correlation between the concentration of MT and Cu or the value of the Cu/Zn ratio.

In both groups of non-smoking and smoking pregnant women we found higher plasma MT concentrations, and almost 4-fold lower plasma SOD activity when compared to non-pregnant non-smoking or smoking women, respectively. SOD is another such parameter associated with Zn and Cu homeostasis [52,53,54]. Lower plasma SOD activity could result from the replacement of Cu from the active center of the enzyme. Also, a higher concentration of Cd can decrease SOD activity, which was earlier claimed in an experimental study by other authors [55]. Moreover, SOD scavenges O_2_^•−^ which is also removed by MT, which could suggest that MT plays a crucial role as an antioxidant during pregnancy. A similar effect of exposure to tobacco smoke on higher MT concentration in the blood of pregnant women with intrauterine growth restriction was observed in our earlier study [1]. In addition, we revealed in a previous study that MT concentration in the first trimester was lower when compared to the third trimester [30]. The kind of exposure to tobacco smoke (passive or active smoking) during pregnancy did not influence MT concentration either in plasma and lysate or plasma SOD activity, whereas exposure to tobacco smoke during pregnancy resulted in a higher MT concentration and SOD activity. We found higher SOD activity in the plasma of pregnant women exposed to tobacco smoke when compared to non-exposed pregnant women, but we did not reveal any significant difference in SOD activity between actively and passively smoking pregnant women. There are fewer data concerning the influence of exposure to tobacco smoke during pregnancy on SOD activity. A previous study has shown a higher SOD activity in the group of smoking pregnant women when compared to non-smoking subjects [56], which was explained by an elevation in Zn concentration, which was also increased in serum of women exposed to tobacco smoke. The same correlation was obtained in the present study, where we found a positive correlation between SOD activity and Zn concentration in the group of actively smoking pregnant women (r = 0.68).

Another important non-enzymatic antioxidant which acts as a major intracellular defense against oxidative stress is GSH [57] which is consumed by this process resulting in lowered intracellular GSH concentration. Also, presumably in our study, higher oxidative stress associated with pregnancy and/or exposure to tobacco smoke significantly decreased the concentration of GSH. Reduced concentration of GSH was also observed by other authors when exposure to tobacco smoke occurs [58] or during pregnancy, which was explained by oxidative stress and increases in plasma volume [59]. Unfortunately, when we evaluated the effect of exposure to tobacco smoke in each trimester of pregnancy in our previous study, we did not reveal any significant differences between non-smoking and smoking pregnant women [30].

It seems that H_2_O_2_ scavenging was dependent on exposure to tobacco smoke. After the conversion of O_2_^•−^ to H_2_O_2_, hydrogen peroxide is decomposed to water by either CAT or GPx enzymes [60]. We found higher activity of CAT only in the group of non-smoking pregnant women when compared to non-smoking non-pregnant women, whereas in other cases we observed significantly lower activity of CAT (in the group of actively smoking pregnant women when compared to actively smoking non-pregnant women or non-smoking and smoking pregnant women (passive or active smoking), which suggests that pregnancy itself is associated with an elevation in CAT activity, whereas exposure to tobacco smoke during pregnancy decreases CAT activity. In contrast, in the group with lower CAT activity, higher GPx activity was observed which confirmed that those two enzymatic antioxidants complemented each other. GPx is the most important enzyme to eliminate peroxides in the cells of mammals and needs GSH as a cofactor which is oxidized in the presence of free radicals [61]. This finding was confirmed in our study by a positive correlation between GSH concentration and GPx activity in the group of non-smoking and actively smoking pregnant women. The results of our study suggest that GPx plays a crucial role as a scavenger of H_2_O_2_ in the group of pregnant women exposed to tobacco smoke and the results are similar to other investigations [56]. Chełchowska et al. also demonstrated, at the beginning of pregnancy, an elevation in activity of GPx, but only in the smoking group, whereas the activity of CAT was increased in the non-smoking group [56].

On the other hand, higher activity of CAT could be associated with a reduced risk of many diseases during pregnancy, when exposure to tobacco smoke did not occur and that decrease in CAT activity during pregnancy in women exposed to tobacco smoke may be indicative of early-onset preeclampsia [36], which confirmed the results of a meta-analysis conducted by Wei et al. [62]. In addition, lower CAT activity in early pregnancy may be related to preterm birth [63], which could be enhanced by exposure to tobacco smoke [11,64]. Probably, in our study lower CAT activity was compensated by higher activity of GPx which should be sufficient to convert H_2_O_2_ to H_2_O [65], even if a higher concentration of Cu occurs.

It is possible that in the first trimester of pregnancy, the antioxidant system is sufficient to prevent oxidative stress, but many diseases develop in the later stages of pregnancy, but to confirm these findings, further investigation on larger sample size is needed.

Our results suggest that MT, SOD, and GPx are important antioxidants during pregnancy when exposure to tobacco smoke occurs, whereas pregnancy itself is associated with a higher concentration of MT and increased activity of CAT. Both exposure to tobacco smoke and pregnancy decrease the concentration of GSH.

Our study also confirmed that active and passive maternal smoking have a similarly negative effect on antioxidant status in the first trimester.

Some limitations should be considered when interpreting these study results. Due to a small number of smoking subjects (11 cases of non-pregnant, active smokers and 14 pregnant active smokers), the obtained results should be treated as a pilot, and should be considered for future studies.

## 5. Conclusions

**Figure 1:**
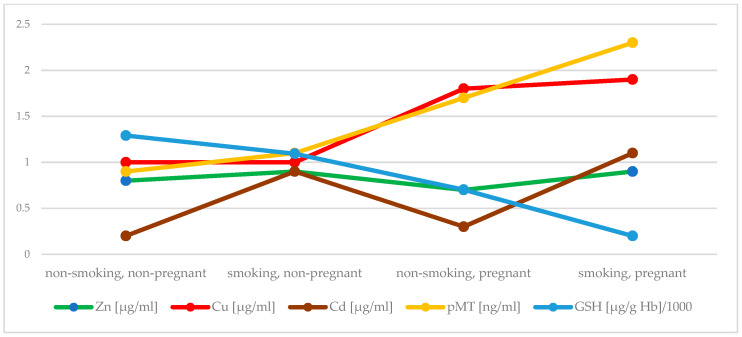
Comparison of the concentartion of Zn, Cu, Cd, MT in plasma and GSH between groups.

**Figure 2:**
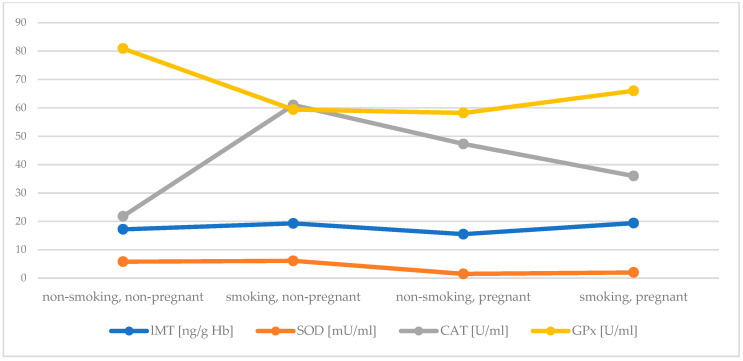
Comparison of the concentartion of MT in lysate and the activity of SOD, CAT and GPx between groups.

Active and passive maternal smoking has a similarly negative effect on antioxidant status in the first trimester. Early pregnancy itself significantly increased serum Cu concentration, while decreased Zn concentration and activity of SOD. In addition, an elevation in activity of CAT in plasma of pregnant women was found, but when exposure to tobacco smoke did not occur. An inverse effect was observed in case of GPx activity. Both investigated factors, i.e., early pregnancy and exposure to tobacco smoke, were related to reduced concentration of GSH. It seems that plasma MT plays a crucial role as an antioxidant during pregnancy (Figure 1 and Figure 2 summarize the main findings).

## Figures and Tables

**Table 1:** Characteristics of studied women.

Parameters	Age (Years)	BMI (kg/m^2^)	Gestational Age (Weeks)	Cotinine (ng/mL)
Non-pregnant non-smoking women	27.0 ± 9.7 (22.0; **22.5**; 26.0)	21.5 ± 2.4 (19.8; **21.3**; 22.5)	Not applicable	2.1 ± 0.8 (0.8; **1.2**; 2.2)
Non-pregnant smoking women	25.5 ± 9.6 (21.0; **23.0**; 25.0)	20.4 ± 2.0 (19.7; **20.7**; 21.2)	Not applicable	81.3 ± 32.3 (55.8; **78.9**; 107.1) *
Pregnant women non-smoking	36.2 ± 3.1 ** (35.0; **36.0**; 38.0)	23.9 ± 3.6 (21.6; **23.6**; 26.2)	12.8 ± 0.6 (12.3; **12.9**; 13.1)	0.6 ± 0.4 (0.4; **0.6**; 0.7)
Pregnant women exposed to tobacco smoke	37.1 ± 2.4 *** (35.0; **36.5**; 39.0)	24.0 ± 3.9 (21.5; **22.8**; 26.3)	12.7 ± 0.6 (12.4; **12.6**; 13.1)	40.0 ± 69.5 (0.5; **1.1**; 46.6) *
Passive, pregnant smokers	36.9 ± 2.4 *** (35.0; **37.0**; 39.0)	22.6 ± 3.0 (20.6; **22.2**; 25.0)	12.5 ± 0.5 (12.1; **12.6**; 12.6)	0.8 ± 0.6 (0.3; **0.6**; 0.7)
Active, pregnant smokers	35.0 ± 4.8 *** (35.0; **35.5**; 38.0)	26.1 ± 3.6 (23.4; **25.6**; 29.1)	12.8 ± 0.6 (12.6; **12.7**; 13.1)	104.5 ± 78.6 (33.8; **94.7**; 197.1) *

* *p* < 0.05 when compared to respectively non-smoking (not exposed) group; ** *p* < 0.05 when compared to non-smoking, non-pregnant women; *** *p* < 0.05 when compared to smoking, non-pregnant women. Values shown as mean ± SD and 1st quartile, **median**, 3rd quartile; BMI—body mass index.

**Table 2:** Values of selected antioxidative parameters, and metals in nonsmokers.

Parameters	No Smokers		
	Non-Pregnant *n* = 52	Pregnant *n* = 98	*p* Value
Zn serum (µg/mL)	0.8 ± 0.1 (0.7; **0.8**; 0.9)	0.7 ± 0.1 (0.6; **0.7**; 0.8)	<0.001
Cu serum (µg/mL)	1.0 ± 0.1 (0.9; **1.0**; 1.0)	1.8 ± 0.4 (1.5; **1.8**; 2.0)	<0.001
Cd whole blood (µg/L)	0.2 ± 0.1 (0.05; **0.1**; 0.2)	0.3 ± 0.2 (0.2; **0.3**; 0.5)	<0.001
Cu/Zn serum	1.2 ± 0.3 (1.0; **1.2**; 1.3)	2.6 ± 0.6 (2.2; **2.6**; 3.0)	<0.001
GSH whole blood (µg/gHb)	1290.9 ± 422.7 (1038.1; **1238.7**; 1424.8)	702.7 ± 404.9 (446.0; **624.7**; 852.6)	<0.001
MT plasma (ng/mL)	0.9 ± 0.5 (0.6; **0.7**; 1.1)	1.7 ± 0.5 (1.3; **1.5**; 2.0)	<0.001
MT lysate (ng/gHb)	17.2 ± 3.1 (14.0; **17.4**; 19.5)	15.5 ± 6.6 (11.0; **14.1**; 17.5)	NS
SOD plasma (mU/mL)	5.8 ± 0.4 (5.6; **5.8**; 5.9)	1.5 ± 0.5 (1.1; **1.6**; 1.9)	<0.001
CAT plasma (nmol/min/mL)	21.8 ± 11.0 (16.0; **17.5**; 25.5)	47.3 ± 30.3 (25.3; **41.8**; 65.0)	0.018
GPx plasma (nmol/min/mL)	80.9 ± 13.0 (75.6; **80.8**; 89.3)	58.2 ± 12.2 (50.1; **55.2**; 62.8)	<0.001

Zn—zinc; Cu—copper; Cd—cadmium; GSH—glutathione; MT—metallothionein; NS—non-significant; SOD—superoxide dismutase; CAT—catalase; GPx—glutathione peroxidase. Values shown as mean ± SD and 1st quartile, **median**, 3rd quartile.

**Table 3:** Values of selected antioxidative parameters, and metals in smokers.

Parameters	Active Smokers		
	Non-Pregnant *n* = 11	Pregnant *n* = 14	*p* Value
Zn serum (µg/mL)	0.9 ± 0.05 (0.9; **0.9**; 0.9)	0.9 ± 0.1 (0.7; **0.9**; 1.0)	NS
Cu serum (µg/mL)	1.0 ± 0.1 (0.9; **1.0**; 1.0)	1.9 ± 0.4 (1.8; **1.9**; 2.1)	<0.001
Cd whole blood (µg/L)	0.9 ± 0.2 (0.7; **0.9**; 1.1)	1.1 ± 0.4 (0.6; **1.0**; 1.6)	NS
Cu/Zn serum	1.1 ± 0.2 (1.0; **1.0**; 1.3)	2.2 ± 0.6 (1.8; **2.1**; 2.4)	<0.001
GSH whole blood (µg/gHb)	1093.4 ± 294.5 (771.3; **1136.5**; 1352.6)	200.0 ± 104.2 (125.8; 200.9; 233.2)	<0.001
MT plasma (ng/mL)	1.1 ± 0.3 (1.0; **1.1**; 1.2)	2.3 ± 1.3 (1.4; **1.9**; 2.6)	<0.001
MT lysate (ng/gHb)	19.3 ± 4.4 (16.1; **19.0**; 21.0)	19.4 ± 15.5 (9.7; **13.4**; 23.8)	NS
SOD plasma (mU/mL)	6.1 ± 0.4 (5.7; **6.0**; 6.4)	2.0 ± 0.7 (1.5; **1.8**; 2.1)	<0.001
CAT plasma (nmol/min/mL)	61.0 ± 15.2 (45.5; **62.5**; 74.0)	36.0 ± 23.4 (19.3; **26.9**; 43.5)	0.005
GPx plasma (nmol/min/mL)	59.4 ± 13.3 (48.5; **57.7**; 73.2)	66.0 ± 22.4 (47.5; **69.6**; 82.4)	NS

Zn—zinc; Cu—copper; Cd—cadmium; GSH—glutathione; MT—metallothionein; SOD—superoxide dismutase; NS—non-significant; CAT—catalase; GPx—glutathione peroxidase. Values shown as mean ± SD and 1st quartile, **median**, 3rd quartile.

**Table 4:** Values of selected antioxidative parameters, and metals in pregnant women non-exposed or exposed (passive or active) to tobacco smoke.

Parameters	Pregnant Women		
	Non-Smoking *n* = 98	Smoking (Passive or Active) *n* = 52	*p* Value
Zn serum (µg/mL)	0.7 ± 0.1 (0.6; **0.7**; 0.8)	0.8 ± 0.1 (0.7; **0.8**; 0.9)	0.028
Cu serum (µg/mL)	1.8 ± 0.4 (1.5; **1.8**; 2.0)	1.8 ± 0.3 (1.6; **1.9**; 2.0)	NS
Cd whole blood (µg/L)	0.3 ± 0.2 (0.2; **0.3**; 0.5)	0.6 ± 0.5 (0.3; **0.5**; 0.9)	0.030
Cu/Zn serum	2.6 ± 0.6 (2.2; **2.6**; 3.0)	2.3 ± 0.5 (2.0; **2.4**; 2.7)	0.027
GSH whole blood (µg/gHb)	702.7 ± 404.9 (446.0; **624.7**; 852.6)	244.2 ± 130.0 (137.6; **227.2**; 296.7)	<0.001
MT plasma (ng/mL)	1.7 ± 0.5 (1.3; **1.5**; 2.0)	2.5 ± 1.4 (1.5; **1.9**; 2.9)	0.029
MT lysate (ng/gHb)	15.5 ± 6.6 (11.0; **14.1**; 17.5)	28.5 ± 21.7 (11.9; **19.8**; 41.8)	0.039
SOD plasma (mU/mL)	1.5 ± 0.5 (1.1; **1.6**; 1.9)	2.1 ± 1.3 (1.4; **1.9**; 2.5)	0.028
CAT plasma (nmol/min/mL)	47.3 ± 30.3 (25.3; **41.8**; 65.0)	32.8 ± 19.0 (19.3; **29.3**; 42.2)	0.047
GPx plasma (nmol/min/mL)	58.2 ± 12.2 (50.1; **55.2**; 62.8)	70.4 ± 18.3 (60.3; **72.2**; 82.4)	0.017

Zn—zinc; Cu—copper; Cd—cadmium; GSH—glutathione; MT—metallothionein; SOD—superoxide dismutase; NS—non-significant; CAT—catalase; GPx—glutathione peroxidase. Values shown as mean ± SD and 1st quartile, **median**, 3rd quartile.

**Table 5:** Values of selected antioxidative parameters, and metals in studied subjects.

Parameters	Exposed to Tobacco Smoke Women		
	Passive *n* = 38	Active *n* = 14	*p* Value
Zn serum (µg/mL)	0.7 ± 0.1 (0.7; **0.7**; 1.0)	0.9 ± 0.1 (0.7; **0.9**; 1.0)	0.004
Cu serum (µg/mL)	1.9 ± 0.2 (1.7; **1.9**; 2.0)	1.9 ± 0.4 (1.8; **1.9**; 2.1)	NS
Cd whole blood (µg/L)	0.3 ± 0.1 (0.2; **0.3**; 0.4)	1.1 ± 0.4 (0.6; **1.0**; 1.6)	<0.001
Cu/Zn serum	2.5 ± 0.5 (2.0; **2.5**; 2.9)	2.2 ± 0.6 (1.8; **2.1**; 2.4)	NS
GSH whole blood (µg/gHb)	250.0 ± 148.3 (130.2; **223.4**; 312.9)	200.0 ± 104.2 (125.8; 200.9; 233.2)	NS
MT plasma (ng/mL)	2.4 ± 1.6 (1.4; **1.8**; 2.8)	2.3 ± 1.3 (1.4; **1.9**; 2.6)	NS
MT lysate (ng/gHb)	19.5 ± 14.0 (9.7; **13.9**; 22.8)	19.4 ± 15.5 (9.7; **13.4**; 23.8)	NS
SOD plasma (mU/mL)	2.0 ± 0.7 (1.4; **1.8**; 2.5)	2.0 ± 0.7 (1.5; **1.8**; 2.1)	NS
CAT plasma (nmol/min/mL)	37.7 ± 20.0 (24.0; **34.2**; 48.3)	36.0 ± 23.4 (19.3; **26.9**; 43.5)	NS
GPx plasma (nmol/min/mL)	65.0 ± 16.8 (53.5; **66.2**; 81.5)	66.0 ± 22.4 (47.5; **69.6**; 82.4)	NS

Zn—zinc; Cu—copper; Cd—cadmium; GSH—glutathione; MT—metallothionein; SOD—superoxide dismutase; NS—non-significant; CAT—catalase; GPx—glutathione peroxidase. Values shown as mean ± SD and 1st quartile, **median**, 3rd quartile.

**Table 6:** Significant correlation coefficients in the group of pregnant women.

Correlation Coefficients		Non-Smoking	Passive Smoking	Active Smoking
MT lysate (ng/gHb)	Cu serum (µg/mL)	0.50 0.036	NS	NS
	Cu/Zn serum	0.59 0.010	NS	NS
GSH (µg/gHb)	GPx plasma (nmol/min/mL)	0.65 0.009	NS	0.58 0.029
	CAT plasma (nmol/min/mL)	−0.44 0.040	−0.49 0.022	NS
	SOD plasma (mU/mL)	NS	NS	0.67 0.012
	Cu/Zn serum	NS	NS	−0.66 0.014
SOD plasma (mU/mL)	Zn serum (µg/mL)	NS	NS	0.68 0.015
GPx plasma (nmol/min/mL)	Cu serum (µg/mL)	NS	−0.64 0.018	NS
CAT plasma (nmol/min/mL)	Zn serum (µg/mL)	NS	−0.62 0.040	NS

Cu—copper; Zn—zinc; GSH—glutathione; MT—metallothionein; SOD—superoxide dismutase; NS—non-significant; GPx—glutathione peroxidase; CAT—catalase.

## Data Availability

The data presented in this study are available on request from the corresponding author.
